# Clinical and ultrasound features of 46 children with suppurative osteoarthritis: experience from two centers

**DOI:** 10.1186/s13018-024-04563-9

**Published:** 2024-04-04

**Authors:** Sai-feng Huang, Yue Teng, Hui-Qing Shi, Wen-Juan Chen, Xue-Hua Zhang

**Affiliations:** 1 Department of Ultrasound, Fujian Children’s Hospital (Fujian Branch of Shanghai Children’s Medical Center), College of Clinical Medicine for Obstetrics & Gynecology and Pediatrics, Fujian Medical University, Fuzhou, 350011 China; 2 Department of Ultrasound, Hunan Children’s Hospital, University of South China, Changsha, 410007 China

**Keywords:** Arthritis, Children, Magnetic resonance imaging, Osteoarthritis, Osteomyelitis, Ultrasonic examination

## Abstract

**Objective:**

Diagnosing musculoskeletal infections in children is challenging. In recent years, with the advancement of ultrasound technology, high-resolution ultrasound has unique advantages for musculoskeletal children. The aim of this work is to summarize the ultrasonographic and clinical characteristics of children with pyogenic arthritis and osteomyelitis. This study provides a simpler and more effective diagnostic basis for clinical treatment.

**Methods:**

Fifty children with osteomyelitis or arthritis were diagnosed via ultrasound, and the results of the ultrasound diagnosis were compared with those of magnetic resonance imaging and surgery. Clinical and ultrasound characteristics were also analyzed.

**Results:**

Out of 50 patients, 46 were confirmed to have suppurative infection by surgical and microbiological examination. Among these 46 patients, 26 were diagnosed with osteomyelitis and 20 had arthritis. The manifestations of osteomyelitis were subperiosteal abscess (15 patients), bone destruction (17 patients), bone marrow abscess (9 patients), and adjacent joint abscess (13 patients). Osteomyelitis mostly affects the long bones of the limbs, femur and humerus (10 and 9 patients, respectively), followed by the ulna, radius, tibia and fibula (one patient each). The manifestations of arthritis were joint pus (20 patients) and joint capsule thickening (20 patients), and hip dislocation (8 patients). All the patients had arthritis involving the hip joint.

**Conclusion:**

Subperiosteal abscess, bone destruction, and joint abscess with dislocation are ultrasonographic features of pyogenic osteoarthritis. The findings of this work can improve the early diagnosis and differentiation of pyogenic osteoarthritis and provide a reliable basis for treatment.

## Background

Diagnosing musculoskeletal infections in children is challenging. Older children may have systemic symptoms, such as fever; infants may only cry, eat poorly, or refuse to move their limbs; and newborns often have no symptoms, such as fever, because of their immature immune system and may even have normal levels of infection and inflammation, including C-reactive protein (CRP) and white blood cell count [1–3]. Although plain X-ray images are easy and inexpensive to obtain, they are insensitive for detecting joint effusion and early osteomyelitis. Magnetic resonance imaging (MRI) is the preferred imaging method because it has good soft tissue contrast and high sensitivity and specificity and can show bone edema early. However, infants and young children need to be deeply sedated prior to MRI. The high risk of radiation exposure limits the use of scintigraphy and computed tomography [4, 5]. Ultrasound is insusceptible to motion and metal artifacts. The structures of unossified cartilage and bone joints in children facilitate ultrasound imaging [6]. Although musculoskeletal ultrasound has been widely used, its application for pediatric diagnosis should be studied in depth. Therefore, this work summarized the ultrasound and clinical characteristics of pyogenic osteoarthritis to provide a correct basis for its clinical diagnosis.

## Materials and methods

### Patient population

This study retrospectively analyzed the general data of 50 patients with purulent osteoarthritis who were diagnosed via ultrasound at Hunan Children's Hospital from January 2016 to April 2019 and Fujian Children’s Hospital from February 2020 to November 2022. Among these patients, 46 were confirmed to have suppurative infection by histopathology and microbiological examination, and four had other causes of infection.

### Laboratory examination

The white blood cell count and C-reactive protein level were recorded.

## Ultrasound

### Ultrasonic equipment

Toshiba Color Doppler Ultrasound Applio 500 and Simens Acuson Sequoia Redwood with linear array probes at a scanning frequency of 7–18 MHz were used on the basis of the condition of the child. Ultrasound images were reviewed and analyzed with the PACS system by pediatric sonographers with more than 5 years of experience.

### Inspection methods

A multisection scan (longitudinal section and cross-sectional scans) of the affected limb was taken, and the skin, subcutaneous tissue, deep fascia, muscle, capsule, joint bursa, joint cavity, periosteum, and bone were assessed. Doppler was used to observe local blood flow in the affected area.

### Radiological examination

Among the patients, 40 underwent MRI of the affected limbs, and six underwent CT.

### Surgery and biopsy

All patients underwent surgery (incision drainage or drainage and joint reduction), and tissue biopsy or pus culture was performed after the operation.

### Groups

Patients were divided into groups according to the results of ultrasonography and surgery.

### Ethical permission

This study was approved by the Medical Ethics Committee of Fujian Children’s Hospital and Hunan Children’s Hospital, and informed consent was waived. All procedures were performed in accordance with the Declaration of Helsinki.

### Statistical methods

SPSS 20.0 was used for statistical analysis. Counting data are expressed as cases and percentages. The median age of the patients was calculated. The Chi-square test was used to compare the sex distributions between the osteomyelitis group and the arthritis group. The Mann‒Whitney test was used to compare the age at onset between patients with osteomyelitis and those with arthritis. The threshold of statistical significance was set at 5%. The agreement between the ultrasound and clinical results was calculated using cross-tabulation.

## Results

### General information

Among the 46 patients with purulent osteomyelitis and arthritis, 21 (45%) were males and 25 (55%) were females. The ages of the patients ranged from 7 days to 13 years and 8 months. In accordance with the initial location and ultrasound manifestations, the 46 patients were divided into the osteomyelitis and arthritis groups. The osteomyelitis group included 26 patients (17 males (65%) and 9 females (35%)), with ages ranging from 9 days to 13 years and 8 months (median age 2 years). Eleven patients complained of limb pain (three patients with fever), eight patients complained of limited limb movement (one patient with fever), and seven patients complained of limb swelling (two patients with fever). Among them, four children with severe disease had bioinflammatory syndrome. The arthritis group included 20 patients aged 7 days to 9 months with a median age of one month; four (20%) were males, and 16 were females (80%). Among these patients, 11 complained of limb dysfunction (with swelling in five patients), seven had limb swelling (with limited mobility in five patients and fever in one patient), and leukocyte elevation was found in one patient due to jaundice.

### Laboratory examination

The white blood cell count increased in 17 patients, and the highest was 35.18 × 10^9/L. CRP levels increased in 26 patients, the highest of which was 180.26 mg/L.

### Radiological examination

The MRI scans of the 40 patients with osteoarthritis showed corresponding infectious lesions of the bone and joint. The CT scans of six patients showed density reduction, periosteal reaction, bone destruction, soft tissue edema and so on.

### Ultrasound results

Among the 46 patients, 26 had osteomyelitis, 21 had a single bone involved, three have two bones invovled, and two had three or more involved bones. Eight patients had lesions involving the humerus, 10 had femoral osteomyelitis (one had multiple femoral osteomyelitis), one had osteomyelitis of the tibia and fibula, three had osteomyelitis of the ulna (two with merged multiple osteomyelitis), two had osteomyelitis of the radius (one with multiple osteomyelitis), and one had osteomyelitis of the heel bone. Ultrasound revealed arthritis in 20 patients, all of which involved the hip joint (including two patients with lesions involving the hip and shoulder joints) (Table 1).

**Table 1:** Frequency distribution of 46 cases of septic osteoarthritis involving bones and joints in this group

Osteomyelitis (26 cases)	*n*	Percentage	Arthritis (20 cases)	*n*	Percentage
Femur (alone)	9	34.6	Hip	18	90
Tibia	4	15.3	Hip + shoulder	2	10
Fibula	1	3.8			
Calcaneus	1	3.8			
Humerus	6	23			
Radius	1	3.8			
Ulna	1	3.8			
Humerus + Ulna	1	3.8			
Brachial + ulnar + radial	1	3.8			
Femur +humerus + Ulna	1	3.8			
Total	26	100	Total	20	100%

#### Osteomyelitis features

In the osteomyelitis group (26 patients), ultrasonography revealed subperiosteal abscess in 15 patients, bone destruction in 17 patients, bone marrow abscess in nine patients, and adjacent joint abscess in 13 patients (Table 2).

**Table 2:** Comparison of findings for 26 patients with osteomyelitis and 20 patients with arthritis

Osteomyelitis(*N* =)	Side	Sex	Median age	Subperiosteal pus accumulation	Bone destruction	Bone marrow vomica	Pyarthrus
26	R = 14 L = 11 Bilateral = 1	M = 17 F = 9	2 y	15	17	9	13
Arthritis	R = 14 L = 6 Bilateral = 0	Sex M = 4 F = 16	1 M	Pyarthrus	Hip dislocation	Hip dysplasia	Bone slightly abnormal
20				20	8	9	10
*Sig*	0.34	0.031*	0.015*				

*indicates a statistically significant difference

Among the patients, two had subperiosteal pus and bone destruction, nine had bone destruction, and three patients had subperiosteal pus and adjacent joint pus, two patients had bone destruction and joint pus, nine patients had subperiosteal pus, one had bone destruction and bone marrow pus, and one patient had subperiosteal pus, one had bone destruction and one had joint pus.

**Fig. 1:**
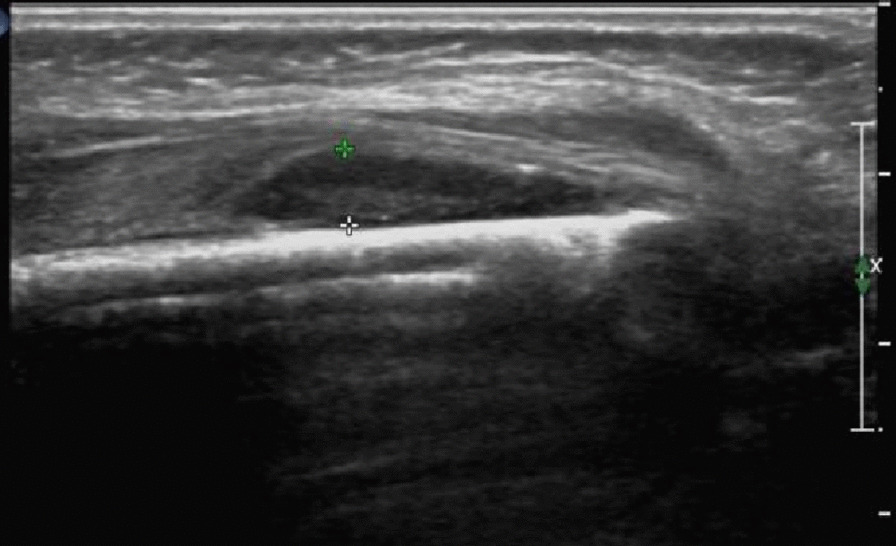
Subperiosteal abscess extending longitudinally along the cortex of long bone

**Fig. 2:**
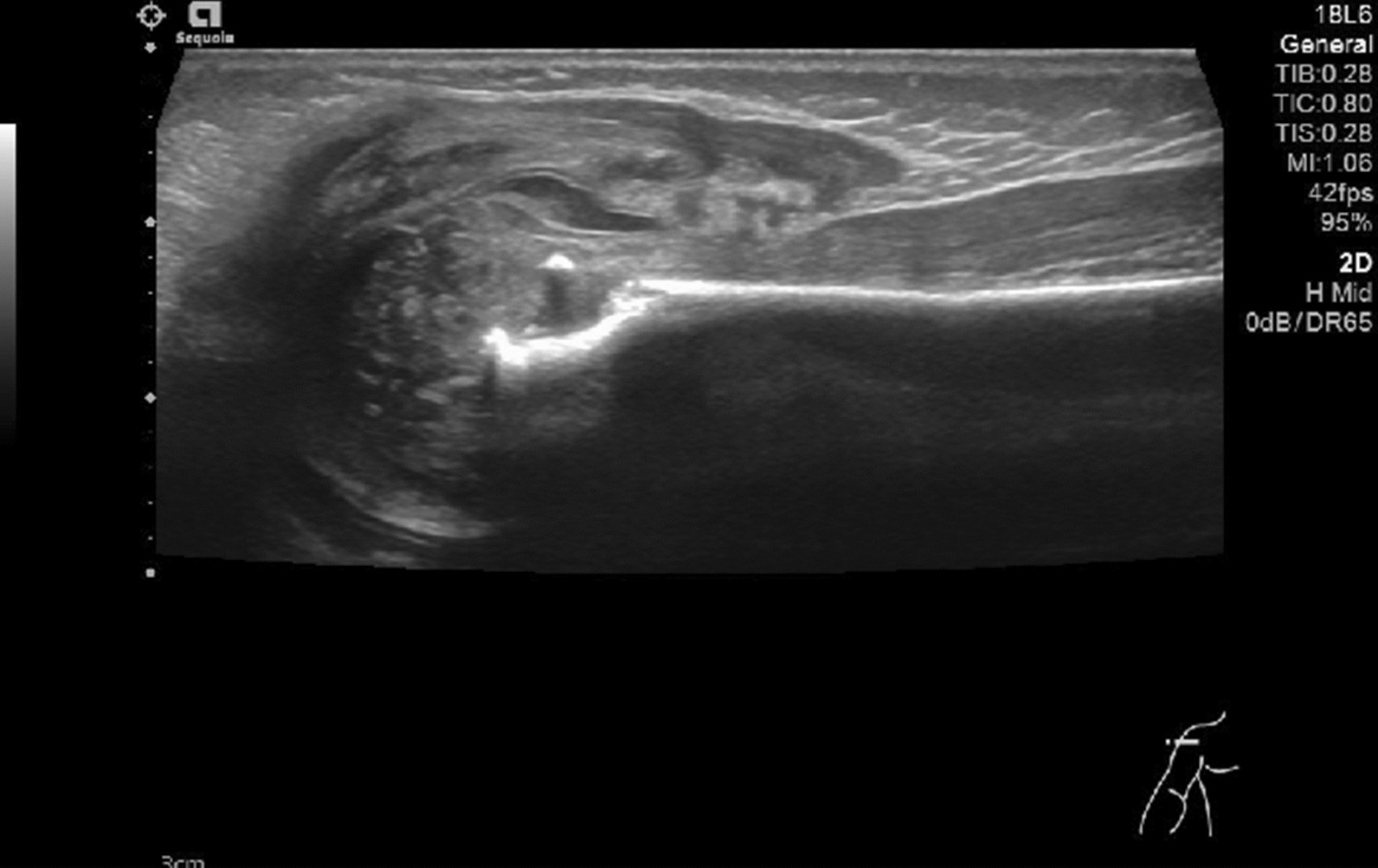
Poor continuity, partial depression and irregular lumpy echo of epiphyseal bone

**Fig. 3:**
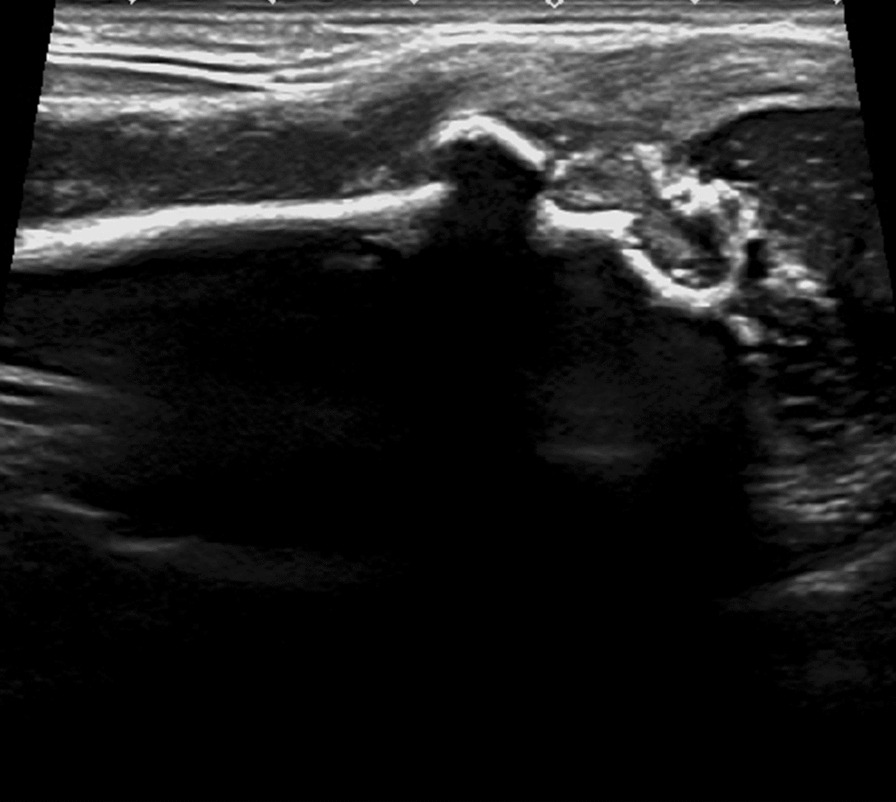
Bone marrow pus cavity of lower femur

**Fig. 4:**
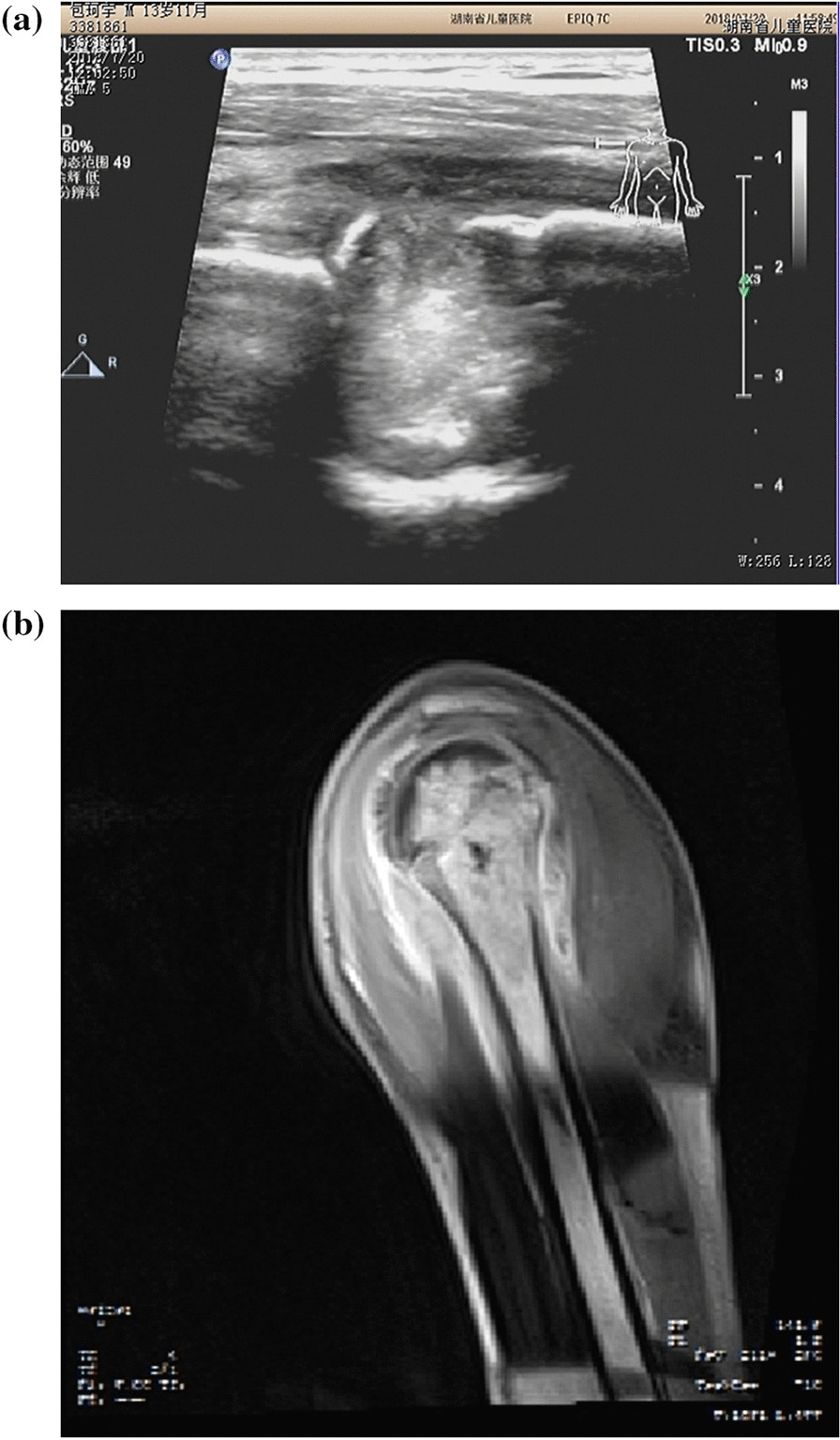
**A** Bone destruction was severe in the epiphysis, local bone invagination and bone marrow abscess cavity were formed, MRI (**B**) showed similar findings (f)

During the early stage, local soft tissue or deep muscle thickening, echo enhancement, and empyema in the deep soft tissue were observed. During the abscess stage, periosteal bulging, thickening, increased tension, and fusiform or crescent-shaped abscesses of subperiosteal abscess were detected (Fig. 1). In the late stage, the bone surface was not smooth, and the bone cortex was continuously interrupted (Fig. 2). In addition, bone collapse and local clumps or fragments were found, indicating bone destruction, dead bone formation   and bone marrow abscess (Fig. 3, 4).

#### Arthritis features

**Fig. 5:**
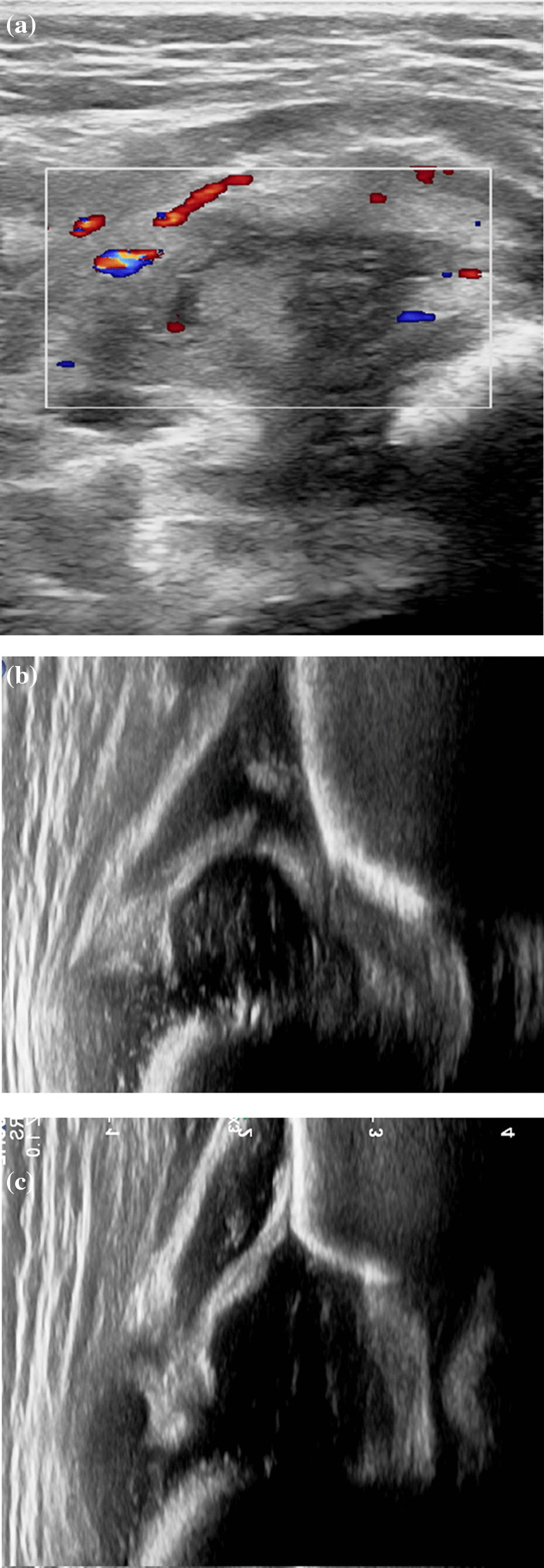
Suppurative coxitis with hip dislocation. **A** Thickening of the hip capsule, fluid accumulation in the joint cavity with poor sound transmission, and prolapse of the femoral head outside the acetabulum (**B**). **C**: The contralateral joint is normal

Ultrasonography revealed that all 20 patients had joint pus and joint capsule thickening, and six patients had mild bone abnormalities (cortical bone thickening and surface roughness) and trace subperiosteal effusion in the proximal femur or neck of the femur. No bone destruction or bone marrow abscess was detected. In contrast, hip arthritis can easily lead to dislocation of the hip joint. Six patients were complicated with dislocation (Fig. 5A–C). Six patients had hip dysplasia (Graf-Ib syndrome) (Table 2)

.

### Pus culture and tissue biopsy

Ultrasound was used to diagnose septic osteoarthritis in fifty patients. Four patients were excluded from this study; one had tuberculosis, two had fungal infection, and one had juvenile idiopathic arthritis (Table 3). Among the patients, 46 were confirmed to have purulent infection by surgery and microbiological examination. Pus culture revealed that eight (17.4%) patients had *Staphylococcus aureus*, four (8.6%) patients had methicillin-resistant *Staphylococcus aureus* (MRSA), two (4.3%) had *Staphylococcus epidermidis*, one (2.2%) patient had *Enterococcus faecalis*, and one (4.3%) patient had a mixed infection, namely, *Pseudomonas aeruginosa* and *Acinetobacter baumannii*(1). There was no growth in 28 (60.8%) patients (antibiotics were used before bacterial culture, and postoperative antibiotic treatment was effective) [7] (Table 3). The biopsy revealed inflammatory changes in the tissues, and some patients who underwent bone tissue extraction showed signs of ischemic necrosis of the bones.

**Table 3:** Joint fluid culture of 50 patients with suspected septic osteoarthritis

Pathogenic bacteria	N
Gram-positive	
*Staphylococcus aureus*	**8**
*Methicillin-resistant Staphylococcus aureus (MRSA)*	**4**
*Staphylococcus epidermidis*	**2**
*Enterococcus faecalis*	**1**
Gram-negative	
*Pseudomonas aeruginosa*	**2**
Mixed infection	
*Pseudomonas aeruginosa and Acinetobacter baumannii*	**1**
None growth	**28**
Other infection	
Tuberculosis	**1**
Fungus	**2**
Juvenile idiopathic arthritis	**1**
**Total**	**50**

### Surgical

Among the 46 patients with suppurative infections, 26 had osteomyelitis, 14 had right limb involvement, 11 had left limb involvement, and one had bilateral suppurative infection. The femur (10 patients, distal femur) was the most commonly affected bone, followed by the humerus (eight patients). Of the 26 patients with osteomyelitis, 13 exhibited adjacent joint inflammation. Twenty patients were diagnosed with suppurative arthritis, including six and 14 patients with right-sided and left-sided suppurative arthritis, respectively. The hip joint was the most common site of arthritis (20 patients, 100%); eight patients exhibited pathological hip dislocation and nine patients exhibited dysplasia (Table 2). Among the 20 patients with hip arthritis, 10 were diagnosed with mild osteomyelitis (proximal femoral osteomyelitis and iliac osteomyelitis) (Table 2).

### Diagnostic consistency analysis

The consistency among the ultrasound, surgical, and microbiological examination results was also analyzed. Compared with surgery and microbiological examination, ultrasound had a sensitivity of 100% (46/46 patients) and a specificity of 91% (46/49 patients) in accurately detecting septic osteoarthritis in children.

Among the 20 patients with hip arthritis, 10 were diagnosed with mild osteomyelitis (proximal femoral osteomyelitis and iliac osteomyelitis), among which 6 had abnormalities in the proximal femur bone on preoperative ultrasound. The other patients were normal. Ultrasound did not reveal proximal femoral osteomyelitis in four patients, and one patient had combined iliac osteomyelitis. One patient with simple shoulder arthritis who was not diagnosed prior to ultrasound surgery subsequently presented with osteomyelitis of the proximal humerus and shoulder arthritis.

## Discussion

This retrospective study revealed that suppurative osteomyelitis and arthritis in children have their own characteristics and that the age at onset, sex, limb involvement, ultrasound manifestations, and clinical complications differ. The osteomyelitis group included more boys than girls, and the arthritis group included more girls than boys. In the osteomyelitis group, the incidences in the left and right limbs were similar. In the arthritis group, most of the patients had lesions in the right limb (14/70%). Most of the individuals with suppurative arthritis were female sex and more likely to have cases involving the right side. Similarly, the incidence of developmental dysplasia of the hip is high in girls [8]. However, whether the mechanism of these diseases is related to estrogen in females is unclear and requires further study. Suppurative arthritis and osteomyelitis involving the right side of the body, especially arthritis associated with hip osteoarthritis, are occasionally associated with ipsilateral arthritis. However, studies with large sample sizes are needed to confirm the characteristics and occurrence rates of these conditions.

The clinical manifestations of the two diseases also differ. Arthritis is more common in younger patients (for a median of one month), and the chief complaint is physical activity limitations. Osteomyelitis has a later onset, and the chief complaint is limb pain. Commonly, although purulent infection occurs, the time of its onset is unknown in young patients because of its low involvement in deep tissue. The indicators of suppurative infection, such as redness, swelling, and fever, are not obvious and thus hinder the clinical diagnosis of these two diseases. In recent years, ultrasonic diagnosis technology has gradually improved. For example, probes with high frequency and resolution can clearly display the states of muscle, tendon, cartilage, and even bone. In this group of patients, 50 were diagnosed with osteoarthropathy according to findings on ultrasound, suggesting suppurative osteomyelitis or arthritis. Although four patients had other infections, 46 patients were confirmed to have suppurative infections. Therefore, ultrasound has high sensitivity and specificity in diagnosing suppurative osteomyelitis or arthritis [9]^.^ It can be used as an effective examination method for the early detection of arthritis. In addition, misdiagnosis or missed diagnoses can easily occur if the scanner is placed too shallow or if the sonographer lacks understanding about the disease. Therefore, the characteristics of suppurative osteomyelitis and arthritis must be summarized to improve the patients’ understanding of the disease and the rate of accurate detection of suppurative osteoarthritis in different periods to therefore avoid missed diagnoses and misdiagnosis.

Pyogenic osteomyelitis can cause direct or hematogenic transmission, and the long bones that are commonly affected by this disease include the femur, tibia, and humerus [4]. In this study, 94% of osteomyelitis cases involved the long bones of the extremities, and the remaining cases involved the epiphysis. The femur was the most commonly involved (10 patients) bone, followed by the humerus. Osteomyelitis can be divided into three stages on the basis of clinical manifestations, namely, acute, subacute, and chronic; these manifestations may overlap in some cases [4]. (1) Subperiosteal abscess manifest during the acute stage (within 1–2 weeks). A total of 15 patients (58%) with osteomyelitis had subperiosteal abscesses, all of which were distributed longitudinally along the long diaphysis bone. Subperiosteal abscesses are characterized by hypoechoic or anechoic effusion extending along the bone cortex, and longitudinal distribution of effusion parallel to the long bone is a characteristic of osteomyelitis [3]. However, whether the presence of effusion between the bone and soft tissues or subperiosteum is important for differentiating osteogenic and nonosteogenic infections remains unclear [3]. (2) Bone destruction is a common manifestation during the subacute stage (10–14 days). A total of 17 patients presented with bone destruction and bone abnormalities. Ultrasound revealed the thickness of the bone, the continuity of the bone cortex, and evidence of bone destruction, such as local depression, flaking, or nodular high echoes. (3) Patients show bone marrow vomica in the chronic stage (after 2–3 weeks). The formation of a purulent cavity in the bone marrow was observed in nine children. The presence of pus and the formation of single or multiple bone marrow vomica are indicative of a local bone defect [3]. Proximal joint pyomedia may occur in subacute and chronic osteomyelitis patients. Eleven patients in this group exhibited significant joint pyomedia, which was secondary to osteomyelitis of the connected bone.

Septic arthritis is a joint infection in which bacteria in the blood spread into the synovium with high vascularity. Bacterial endotoxins, inflammation, and nutrient-packed vessels caused by a large amount of fluid in the joints cause avascular necrosis of the joint, which leads to joint damage and thus requires rapid diagnosis and early treatment [5, 6]. Fluid first accumulates in different areas of each joint: the suprapatellar capsule of the knee joint, the front recess of the hip joint, or the posterior recess of the elbow joint [3]. The hip joints are most affected in this group of arthritis patients. The characteristics of septic hip arthritis (18 patients with monoarthritis and two patients with combined shoulder arthritis) were retrospectively analyzed: (1) septic hip arthritis was more common in newborns (100% of patients under 1 year old with a median age of 1 month). (2) The ultrasound features included poorly transmitted fluid in the joint cavity and around the femoral head, a thickened joint capsule, thickening and edema of extracapsular soft tissue edema, and a short femoral neck. Anterior crypt effusion, which can be distinguished from transient synovitis in the anterior crypt or iliopsoas muscle bursa [10], was also observed. (3) The femoral head and metaphysis were involved, resulting in slight bone changes. A review of the case images showed that 10 out of the 20 patients with hip arthritis in this group exhibited changes in the femur with mild osteomyelitis during the operation. However, no obvious bone damage or changes were found during the operation. (4) Pathological dislocation of the hip can easily occur in patients with hip arthritis. The pus passed through the hip joint, and the joint capsule became swollen and loose. In addition, hip dislocation is related to inflammation, which affects the blood supply and development of the femoral head. Ultrasound revealed that the femoral head on the diseased side was smaller than that on the contralateral side during preoperative and postoperative follow-up examination. Delayed ossification may be another cause of hip dislocation. However, given the lack of long-term follow-up observations of multiple samples, further research is needed for confirmation.

## Conclusion

Joint abscess with dislocation, subperiosteal abscess, bone destruction, and abscess in the bone marrow cavity are the ultrasonic characteristics of suppurative osteoarthritis. Ultrasound can be used to diagnose and differentiate these diseases early. This imaging test, in combination with clinical characteristics, can provide a reliable basis for the diagnosis and treatment of suppurative osteoarthritis.
